# Combined Cytogenotoxic Effects of Bee Venom and Bleomycin on Rat Lymphocytes: An *In Vitro* Study

**DOI:** 10.1155/2014/173903

**Published:** 2014-04-16

**Authors:** Yasmina M. Abd-Elhakim, Samah R. Khalil, Ashraf Awad, Laila Y. AL-Ayadhi

**Affiliations:** ^1^Department of Forensic Medicine and Toxicology, Faculty of Veterinary Medicine, Zagazig University, Zagazig 44511, Egypt; ^2^Animal Wealth Development Department, Faculty of Veterinary Medicine, Zagazig University, Zagazig 44511, Egypt; ^3^Physiology Department, Faculty of Medicine, King Saud University, Riyadh, Saudi Arabia; ^4^AL-Amodi Autism Research Chair, Autism Research and Treatment Centre, Riyadh, Saudi Arabia

## Abstract

This study was carried out to determine the cytotoxic and genotoxic effects of bee venom (BV) and/or the chemotherapeutic agent bleomycin (BLM) on healthy isolated rat lymphocytes utilizing morphometric and molecular techniques. Using the Ficoll-Histopaque density gradient centrifugation technique, lymphocytes were isolated, divided into groups, and subjected to BV and/or BLM at incubation medium concentrations of 10 or 20 **μ**g/mL respectively for 24 and 72 hrs. An MTT assay and fluorescent microscopy examinations were used to assess the cytotoxic effects. To determine the predominant type of BV and/or BLM-induced cell death, LDH release assay was employed beside quantitative expression analyses of the apoptosis-related genes (Caspase-3 and Bcl-2). The genotoxic effects of the tested compounds were evaluated via DNA fragmentation assay. The results of these assays demonstrated that BV potentiates BLM-induced cytotoxicity through increased LDH release and diminished cell viability. Nevertheless, BV significantly inhibited the BLM-induced DNA damage. The results verify that BV significantly attenuates the genotoxic effects of BLM on noncancerous isolated rat lymphocytes but does not diminish BLM cytotoxicity.

## 1. Introduction

In recent decades, cancer has become one of the most common causes of death, and it creates enormous health and economic issues [1]. Unfortunately, despite the increasingly curative role of chemotherapy in the treatment of various cancers, most chemotherapeutic agents are not precise or specific enough to distinguish between neoplastic and healthy cells; therefore, genotoxicity of chemotherapy often affects both neoplastic and healthy cells equally [2], which may result in the generation of secondary malignancies [3]. The anticancer effects of many natural products have been documented [4].

Honeybee (*Apis mellifera*) venom (BV) tops the list of the newly introduced natural anticancer agents [5]. The radioprotective, antimutagenic, pain-relieving, anti-inflammatory, antiarthritic, antihyperalgesic, and antinociceptive effects of BV have been documented [6–8].

BV is a highly complex blend of at least 18 active components, including active peptides, enzymes, and amines that contain histamine, catecholamines, polyamines, melittin, and phospholipase A_2_ [9]. Melittin, a highly basic polypeptide, is the principal component of BV and constitutes approximately 50% of its dry weight [10].

Several studies have demonstrated that BV and/or melittin have direct antitumor effects* in vivo* [11–13] and* in vitro* on various types of cancerous cells, including liver [14], bone [15], prostate, breast, cervical, and renal cancer cells [16, 17]. Moreover, these studies cited several mechanisms of BV cytotoxicity, including cell cycle alterations, effects on proliferation and/or growth inhibition, and induction of apoptosis or necrosis [18].

Despite this vast number of studies concerned with the various mechanisms of BV cytotoxicity on cancer cells, only very few reports are available on its effects on normal cells [19] and its potential to alter the cytogenetic toxicity effects of anticancer antibiotics in normal cells.

Various drugs have been used to reduce the risk of development of a variety of tumors through regulation of apoptosis [20], including the chemotherapeutic agent bleomycin (BLM). BLM is a water-soluble antibiotic and a key element in the gold standard chemotherapy regimens that are typically used in the treatment of lymphomas and carcinomas [21]. Nevertheless, 46% of cases treated with BLM-containing chemotherapy regimens suffer from various degrees of pulmonary toxicity [22].

The process of apoptosis has been demonstrated to be the primary mode of cell death in resting and cycling human lymphocytes exposed to BLM [23]through Caspase-8 activation, suggesting the involvement of the extrinsic pathway of apoptosis [24].

Previously studied cancer cell lines have illuminated the mitigating effect of BV on the adverse effects of BLM [25].However, little is known regarding the combined effects of BV and BLM on healthy isolated lymphocytes. Therefore, the aim of this study was to evaluate the cytotoxicity (MTT assay, LDH release percentage, fluorescent microscopy examinations, and a quantitative expression analysis of the apoptosis-related genes “Caspase-3 and Bcl-2”) and genotoxicity (DNA fragmentation assay) of BV and its role in the modulation of BLM-induced cellular alterations.

## 2. Materials and Methods

### 2.1. Animals

Adult male Sprague-Dawley rats (120–150 g) were used in this study. They were obtained from the Laboratory Animal farm of the Faculty of Veterinary Medicine of Zagazig University and acclimated to the laboratory environment for 2 weeks prior to use. The animals were housed in stainless-steel cages, maintained in a 12 h light-dark cycle at a controlled temperature (21–24°C) and relative humidity (50–60%), and given standard diet and water* ad libitum* throughout the study. The care and welfare of the animals conformed to the guidelines of the Animal Use Research Ethics Committee of Cairo University, Egypt.

### 2.2. Tested Compounds and Chemicals

Dried pure Egyptian honeybee venom* (Apis mellifera lamarckii)* was obtained and identified according to Schmidt [26] by the Bee Research Department, Plant Protection Institute, Ministry of Agriculture, Egypt. BLM was purchased from Nippon Kayaku Co. Ltd. (Tokyo, Japan). All other reagents, chemicals, and culture media used were of analytical grade and were purchased from the Sigma-Aldrich Co. (St. Louis, MO, USA).

### 2.3. Preparation of Isolated Rat Lymphocytes

Whole blood samples were collected in heparinized tubes from the retro-orbital venous plexus through the medial canthus of the eye from light ether anesthetized rats. Peripheral lymphocytes were isolated using the Ficoll-Histopaque density gradient centrifugation technique according to M'Bemba-Meka et al., [27]. After collection, the blood was diluted 50% with balanced phosphate-buffered saline (PBS). The diluted blood samples were layered on top of Histopaque 1077 (Ficoll/sodium diatrizoate) and centrifuged at 400 ×g for 30 minutes at room temperature. The mononuclear interphase layer was taken and washed three times with Hank's Balanced Salt Solution (300 ×g, 10 minutes). Following the last wash, the cells were counted and resuspended in RPMI-1640 media, pH 6.8, containing 25 mM Hepes, 15 *μ*g/mL phytohemagglutinin, 10% fetal bovine serum, 100 U/mL penicillin, and 0.1% streptomycin to obtain a final concentration of 1 × 10^6^ cells/mL. Each freshly prepared cell suspension had 90% or greater viability prior to each experiment.

### 2.4. Cultivation and Treatment of Isolated Peripheral Rat Lymphocytes

Isolated lymphocytes were cultivated in a sterilized 96-well tissue culture plate and immediately treated with BV and/or BLM dissolved in RPMI-1640 media to obtain the final concentration (10 *μ*g and 20 *μ*g/mL of RPMI media, resp.). These concentrations were chosen based upon a preliminary study with different concentrations of both BLM and BV (Data not shown). Control cells were treated only with RPMI 1640. The cells were then incubated for 24 and 72 hrs at 37°C with 5% CO_2_. The cells were then collected and used for assessment.

### 2.5. Lymphocyte Proliferation Assay

The lymphocyte replication rate was determined using the colorimetric lymphoproliferative assay using MTT dye (3-[4,5-dimethylthiazol-2-yl]-2,5-diphenyl-tetrazolium bromide) according to Bounous et al. [28]. The assay was carried out in a 96-well microculture plate and the optical density (OD) was measured spectrophotometrically after 4 hours at wavelength 570 nm. The replication index (RI) was calculated by applying the following formula: RI = OD at 24 hr or 72 hr/OD at 0 hr. The viability percentage was calculated as follows: (the absorbance of treated groups/the absorbance of control group) × 100.

### 2.6. Lactate Dehydrogenase (LDH) Release Assay

Lymphocytes were evaluated for the presence of necrotic cell death by measuring LDH release from cells into the culture medium [29]. LDH release was quantified using ready-made detection kits from Spinreact, S.A. (Ctra. Santa Coloma, Spain).

### 2.7. Lymphocyte Morphological Changes (Fluorescent Microscopy)

Fluorescent microscopy examinations were employed in this study to assess the morphological changes of isolated lymphocytes [30]. A total of 200 *μ*L from the control and treated isolated lymphocytes was incubated with 2 *μ*L acridine orange and ethidium bromide (in PBS) to visualize apoptotic cells. The cells were viewed using an OPTIKA, B-353LD1, LED-Fluorescence Microscope at 20x using FITC filter sets to excite (488 nm) and visualize (530 nm) stains.

### 2.8. DNA Fragmentation Assay

For quantification of DNA cleavage, the DNA fragmentation assay was performed using a diphenylamine colorimetric assay [31, 32]. Briefly, treated cells were centrifuged at 300 g for 10 min to separate the supernatant from cell pellets. One hundred microliter aliquots of supernatants (designated as* S*) were used for DNA fragmentation assay. The cell pellets were lysed with 0.5 mL of a hypotonic solution containing 10 mM Tris HCl, pH 7.4, 1 mM EDTA, and 0.2% Triton X-100. The lysate was centrifuged for 10 min at 13,000 ×g to separate high molecular weight, intact chromatin (pellet, designated as* B*) from cleaved, and low molecular weight DNA (top solution, designated as* T*). The supernatant (*S*), low molecular weight (*T*) fractions and high molecular weight (*B*) fractions were precipitated overnight with 0.5 mL of 25% trichloroacetic acid (TCA), sedimented at 13,000 ×g for 10 min, and hydrolyzed in 80 mL of 5% TCA at 90°C for 15 min. All fractions were incubated with 160 mL of the diphenylamine reagent at 37°C for 4 hrs, in which 150 mg of diphenylamine was dissolved in 10 mL of glacial acid; then 150 mL of concentrated sulfuric acid and 50 mL of acetaldehyde solution were added and mixed well. The amount of DNA in each sample was estimated from its absorbance at 570 nm in a plate spectrophotometer. The results were calculated using the following equation:

(1) $$\begin{array}{c} \%\;\text{DNA}\;\text{fragmentation}\;=\;\left[\frac{\left(S+T\right)}{\left(S+T+B\right)}\right]*100, \end{array}$$

where* S* is the amount of DNA in the supernatant,* T* the amount of low molecular weight cleaved DNA in the top solution, and* B* the amount of high molecular weight, intact chromatin DNA.

### 2.9. Expression of Apoptosis-Related Genes (Caspase-3 and Bcl-2)

#### 2.9.1. Total RNA Extraction and cDNA Synthesis

Total RNA was extracted from control and treated lymphocytes using the GeneJET RNA Purification kit (Fermantus, UK) following the manufacturer's protocol. The concentration and the integrity of the RNA were assessed spectrophotometrically at 260/280 nm ratio and by gel electrophoresis, respectively. The first-strand cDNA was reverse-transcribed from 1 *μ*g of total RNA using a Quantitect Reverse Transcription kit (Qiagen, Germany) in accordance with the manufacturer's instructions. These samples were subsequently frozen at −80°C until they were used for the determination of the expression level of Caspase-3, Bcl-2, and *β*-actin genes using real-time PCR.

#### 2.9.2. Quantitative Real-Time-PCR

Quantitative real-time PCR was performed on a Rotor-Gene Q cycler (Qiagen, Germany) using QuantiTect SYBR Green PCR kits (Qiagen, Germany) and forward and reverse primers for each gene. The sequences of the primers were as follows: Caspase-3 (NM_012922.2), forward primer: 5′-AAT TCA AGG GAC GGG TCA TG-3′, reverse primer: 5′-GCT TGT GCG CGT ACA GTT TC-3′, Bcl-2 (AF512835.1), forward primer: 5′-TTG ACG CTC TCC ACA CAC ATG-3′, reverse primer: 5′-GGT GGA GGA ACT CTT CAG GGA-3′ and *β*-actin (NM_031144.3), forward primer: 5′-TTG CTG ATC CAC ATC TGC TG-3′, and reverse primer: 5′-GAC AGG ATG CAG AAG GAG AT-3′. The real-time PCR mixture consisted of 12.5 *μ*L 2x SYBR Green PCR Master Mix, 1 *μ*L of each primer (10 pmol/*μ*L), 2 *μ*L cDNA, and 8.5 *μ*L RNase free water in a total volume of 25 *μ*L. The amplification conditions and cycle counts were 95°C for 15 min for the initial activation, then 40 cycles of denaturation at 94°C for 15 s, annealing at 60°C for 30 s, and elongation at 72°C for 30 s. Melting curves were performed after real-time PCR to demonstrate the specific amplification of single products of interest. A standard curve assay was performed to determine the amplification efficiency of the used primers. For each group at the 24 and 72 hr after treatment time points, three independent samples were assessed.

Relative fold changes in the expression of target genes (Caspase-3 and Bcl-2) were accomplished using the comparative 2^−ΔΔCt^ method [33] with the *β*-actin gene as an internal control to normalize the level of target gene expression. ΔΔCT is the difference between the mean ΔCT (treatment group) and mean ΔCT (control group), where ΔCT is the difference between the mean CT gene of interest and the mean CT internal control gene in each sample. Logarithmic transformation was performed on fold change values before being statistically analyzed, using the fold change values of three replicates for each gene measured.

### 2.10. Data Analysis

The results were evaluated using one-way analysis of variance (ANOVA) followed by Duncan's Multiple Range test [34]. Data were expressed as the mean ± standard error (SE). The 0.05 level of probability was used as the criterion for significance.

## 3. Results

### 3.1. Effects of BV and/or BLM on Cell Viability and Proliferation

**Figure 1:**
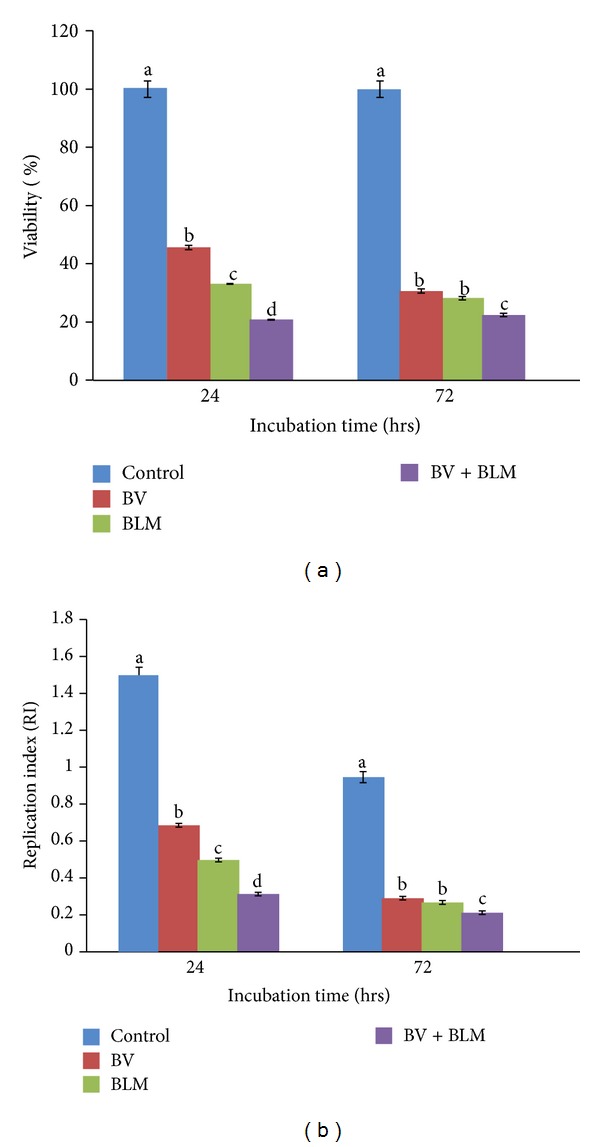
Viability percentage (a) and replication index (b) after* in vitro* treatment of rat peripheral blood lymphocytes with BV (10 *μ*g/mL of RPMI media) and BLM (20 *μ*g/mL of RPMI media) and their combination for 24 or 72 hrs of incubation. Data are expressed as the mean ± S.E. (*n* = 5 replicates). Bars carrying different superscripts at each time point are significantly different (one-way ANOVA) (*P* ≤ 0.05).

Cell survival was diminished significantly in all treated groups relative to the control at both time points of incubation (24 and 72 hrs), but BLM exceeded BV potency in killing normal lymphocytes by 12.48% at the first time point and this gap was reduced to 2.48% at the second time point. The antiproliferative properties of both BV (0.685 ± 0.01; 0.290 ± 0.01) and BLM (0.497 ± 0.00; 0.267 ± 0.01) were evident in comparison to the control (1.501 ± 0.04; 0.946 ± 0.03) at both time points of incubation, but BLM and their combination were more prominent than BV alone (Figures 1(a) and 1(b)).

### 3.2. Effects of BV and/or BLM on Lactate Dehydrogenase (LDH) Release

**Figure 2:**
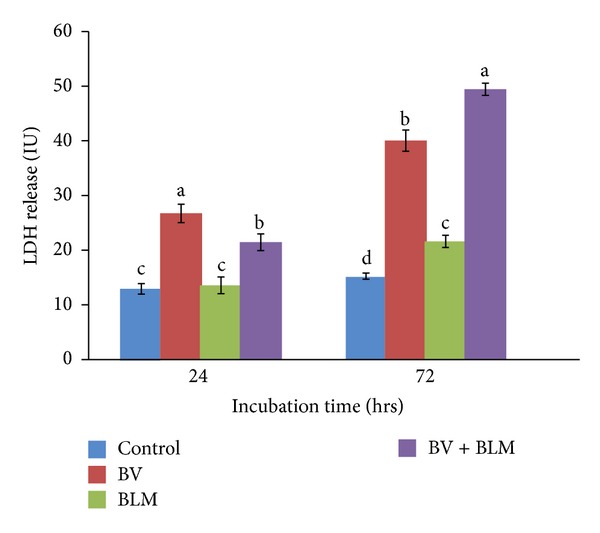
LDH release after* in vitro* treatment of rat peripheral blood lymphocytes with BV (10 *μ*g/mL of RPMI media) and BLM (20 *μ*g/mL of RPMI media) and their combination for 24 or 72 hrs of incubation. Data are expressed as the mean ± S.E. (*n* = 5 replicates). Bars carrying different superscripts at each time point are significantly different (one-way ANOVA) (*P* ≤ 0.05).

After 24 hr of incubation, BV-treated lymphocytes exhibited markedly significant elevations in LDH release (26.73 ± 1.67, *P* ≤ 0.05), while BLM treated replicates showed non-significant (13.55 ± 1.53) increases; however, at co-exposure to both BV and BLM, LDH release increased significantly (21.45 ± 1.65) in comparison to the control group (12.93 ± 0.97) (Figure 2). After 72 hr of incubation, all treated lymphocytes released significantly more LDH relative to the control group, but BV caused the highest LDH values.

### 3.3. Morphological Changes of BV and/or BLM Treated Lymphocytes

**Figure 3:**
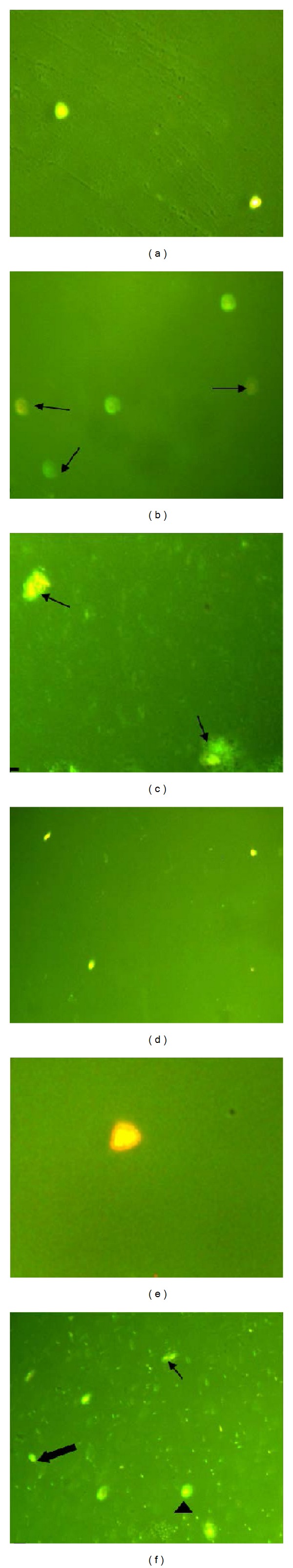
Cell viability microphotographs represent (a) viable untreated rat peripheral blood lymphocytes, (b) and (c) cells with lytic membranes and others in advanced stages of necrosis from the sample treated with BV (10 *μ*g/mL), (d) and (e) apoptotic and dead cells from the sample treated with BLM (20 *μ*g/mL), and (f) viable (arrow head), apoptotic (thick arrow), and necrotic (thin arrow) lymphocytes from samples treated with both BV and BLM.

Fluorescence microscopy examinations of rat lymphocytes subjected to BV showed typical morphological features of necrosis, including lytic cell membranes and ruptured cells (Figures 3(b) and 3(c)); by contrast, apoptotic cellular events were the prominent feature in the BLM treated group and included chromatin condensation, cell shrinkage, and cell death (Figures 3(d) and 3(e)). The BV and BLM cotreated group contained different cells forms, including normal, necrotic, and apoptotic cells (Figure 3(f)).

### 3.4. Effects of BV and/or BLM on DNA Fragmentation

**Figure 4:**
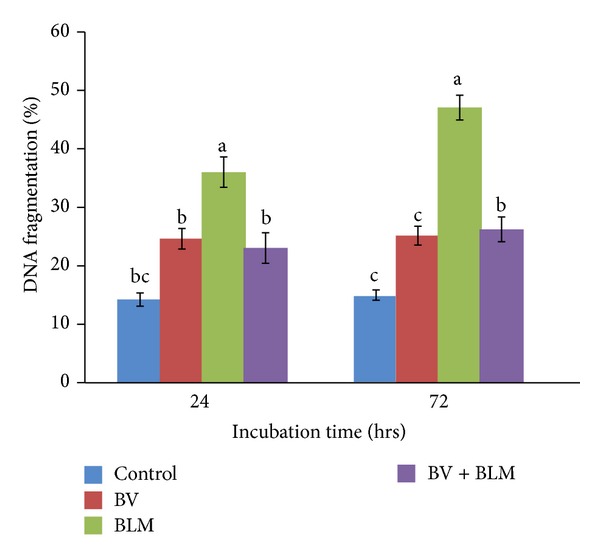
DNA fragmentation percentage of rat peripheral blood lymphocytes after* in vitro* treatment with BV (10 *μ*g/mL of RPMI media) and BLM (20 *μ*g/mL of RPMI media) and their combination for 24 or 72 hrs of incubation. Data are expressed as the mean ± S.E. (*n* = 5 replicates). Bars carrying different superscripts at each time point are significantly different (one-way ANOVA) (*P* ≤ 0.05).

Cells exposed to BV alone did not display any significant increase in fragmented DNA at both time points compared with the untreated cells at *P* ≤ 0.05 (Figure 4). However, BLM treated lymphocytes exhibited markedly significant elevations in DNA fragmentation (36.01 ± 2.60) at 24 hr after treatment, and these elevations were significantly higher at 72 hr (47.03 ± 2.13) in comparison to the control group. When the chemotherapeutic BLM was cotreated with BV, DNA fragmentation was reduced significantly to (23.03 ± 1.76) and (26.24 ± 1.15) at 24 and 72 hr of incubation, respectively.

### 3.5. Effects of BV and/or BLM on Caspase-3 and Bcl-2 Expression Levels

**Figure 5:**
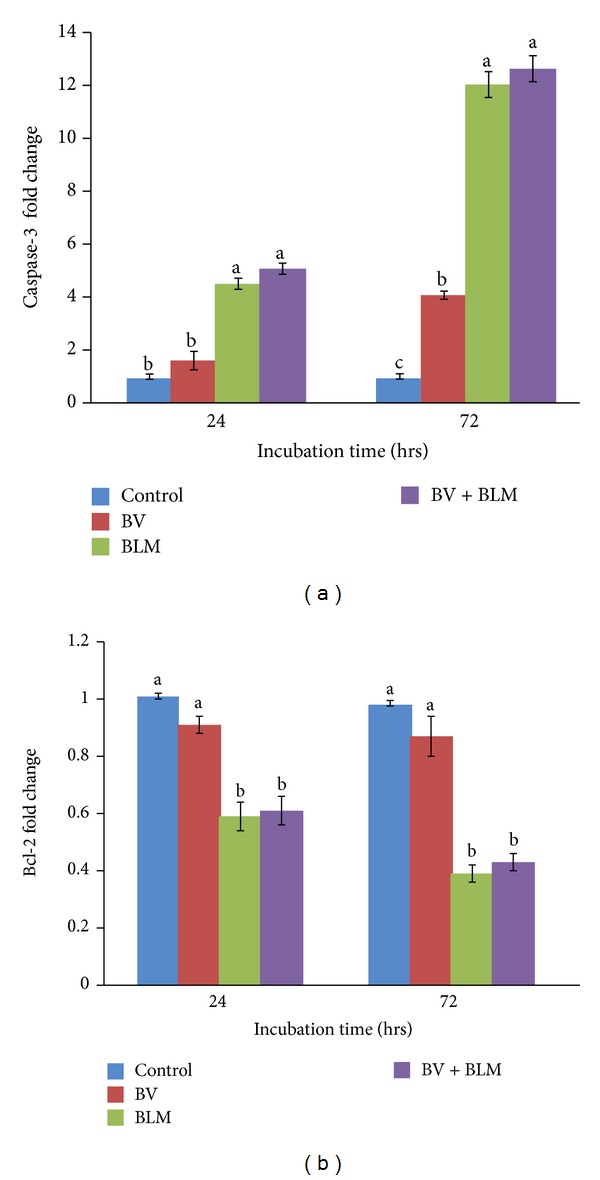
Quantitative RT-PCR analysis of the fold change of Caspase-3 (a) and Bcl-2 (b) at various time points after different treatments (relative to control). Data are expressed as the mean ± S.E. (*n* = 3 replicates). Bars carrying different superscripts at each time point are significantly different (one-way ANOVA) (*P* ≤ 0.05).

As shown in the BLM treated group, the expression of Caspase-3 was significantly upregulated in the initial 24 hr relative to the controls (4.50 ± 0.35-fold; *P* ≤ 0.05); then, the levels sharply increased at 72 hr after treatment (12.03 ± 0.15-fold) (Figure 5). In the BLM plus BV group, Caspase-3 expression was similar to that in the BLM-only group at both time points. In the BV treated group, however, there was a non significant increase in Caspase-3 expression (1.60 ± 0.21-fold, similar to the control value) at 24 hr after treatment and expression became significantly higher at 72 hr (4.07 ± 0.49-fold) in comparison to the control group. However, in the BLM treated group and the BLM plus BV treated group, the expression of Bcl-2 was significantly decreased at 24 hr compared to the control group (0.59 ± 0.05 and 0.61 ± 0.05-fold, respectively, *P* ≤ 0.05). Additionally, this expression was downregulated at 72 hr after treatment (0.39 ± 0.03 and 0.43 ± 0.02-fold, resp.) relative to the controls. However, in BV treated lymphocytes, Bcl-2 expression showed non significant downregulation at 24 hr after treatment (0.91 ± 0.03-fold), which was sustained at nearly the same level at 72 hr after BV treatment (0.87 ± 0.07-fold) compared to the control group.

## 4. Discussion

Antineoplastic drugs are routinely used to combat different types of cancer; yet most of these drugs have unwanted genetic effects on healthy cells and undesirable side effects [35]. BLM is a key component of curative chemotherapeutic regimens used in the treatment of various malignancies. However, BLM increases the risk for a life threating lung injury and a wide variety of chromosomal aberrations in noncancerous cells [21]. In addition to its diverse curative applications, BV has been proven to be a useful compound in potentiating the apoptotic killing capacity of some chemotherapeutic agents such as BLM in cancer cell lines [25, 36]. However, Park et al. [37] recorded the antiapoptotic effect of BV against tumor necrosis factor (TNF)-*α* with actinomycin D-induced apoptosis in normal hepatocyte cell lines. Hence, the purpose of the current study was to determine whether BV has the same effect as BLM on normal isolated lymphocytes.

To determine the effects of BV on lymphocytes, several techniques were adopted as sensitive methods for the evaluation of cytogenetic alterations [30]. An MTT reduction assay and fluorescent microscopy examination were used to evaluate cell survival, whereas the type of cell death was determined using LDH release assay, key signature of necrosis, and the expression level of apoptotic protein Caspase-3 and the antiapoptotic gene Bcl-2. The genotoxic effects were evaluated using the DNA fragmentation assay.

In the present study, the evaluation of cytotoxicity by an MTT assay revealed that BV triggered the cytotoxic and antiproliferative effects of BLM on noncancerous isolated rat lymphocytes. The same result was observed in Chinese hamster lung fibroblasts subjected to cotreatment with BV and BLM [25]. Moreover, the inhibitory impact of BV solely on cell viability and proliferation was observed in several types of nontumor isolated cells (i.e., normal human lymphocytes) and cell lines (human embryonic kidney HEK-293 cells and normal Hef fibroblasts), as well as tumor cell lines (human laryngeal HEp-2 and cervical carcinoma HeLa cells and their drug resistant sublines, breast adenocarcinoma MCF-7 cells, colon adenocarcinoma SW620 cells, and glioblastoma A1235 cells) [38, 39]. However, Kim et al. [40] noted that the viability of an uninjured mouse hepatocyte cell line treated with BV did not change significantly, although this was most likely due to the minute concentration used (10 ng/mL). Similarly, Garaj-Vrhovac and Gajski [41] declared that high concentrations of BV (100 *μ*g/mL) led to cellular instability in human lymphocytes.

The aforementioned cytotoxic enhancement of BLM via BV could possibly be attributed to the anticalmodulin activity of the major BV component melittin, through the formation of a calcium-dependent high-affinity complex of calmodulin and melittin [42]. Calmodulin plays a vital role in normal cell functions, such as extracellular calcium pumping via calcium-magnesium ATPase,microtubule performance, and activation of many intracellular enzymes, including protein kinases, phosphatases, and cyclic nucleotide phosphodiesterase [43]. In addition, both calcium and magnesium play remarkable roles in eliciting cell proliferation in normal cells but not in cancer cells [44].

Apoptosis was shown to be the form of BV-induced cell death in some cell-like synovial fibroblasts and vascular smooth muscle cells [45, 46], while novel studies had described the necrosis of fibroblast-like synoviocytes, human cervical carcinoma HeLa cells, and Chinese hamster lung V79 fibroblasts upon exposure to BV [25, 47]. In this study, most isolated lymphocytes subjected to BV were undergoing very little apoptosis and predominantly necrosis, as indicated by the superabundant release of LDH and morphological analyses with fluorescence microscopy. These effects could be mainly attributed to the amphipathic properties of the melittin peptide that enable it to harm cell membrane bilayer integrity, by either defecting, disrupting, or forming pores in it [47]. Additionally, another constituent in BV, phospholipase A2, catalyzed the hydrolysis of membrane glycerophospholipid bonds, disrupting membrane integrity by releasing lysophospholipids and fatty acids, which themselves may further damage the membrane [48].In contrast, BLM was a very weak LDH releaser compared to BV, which supports the previous findings, confirming that plasma membrane perturbations by BLM are not correlated with its cytotoxicity [49].

In order to evaluate the genotoxic effects induced by BLM-BV cotreatment, DNA fragmentation was analyzed. Primarily, BLM alone generated an abundance of DNA fragments, but when combined with BV, a pronounced reduction in the percentage of DNA fragments was recorded. Similar results were noted in normal human lymphocytes [40]. However, Garaj-Vrhovac and Gajski [41] indicated that high concentrations of BV can lead to DNA damage. The potentially reduced genotoxicity of BV in normal cells may be attributed to the high reactivity of BV against cell membrane that results in cell killing without reaching the DNA and exerting genotoxic effects and also the potential of BV to activate the forkhead subfamily transcription factors FKHR and FKHR1, which have been proven to have the ability to repair damaged DNA [38, 47].

Although there are many features of apoptosis, its hallmark is the expression of groups of gene known as caspases and their regulation by antiapoptotic proteins such as Bcl-2 [50]. Caspase-3 in particular is one of the key executioners of apoptosis and is either partially or wholly responsible for the proteolytic cleavage of many proteins [51], and Bcl-2 represents an evolutionarily conserved apoptotic regulator.

Therefore, in the present context, the expression of Caspase-3 gene and antiapoptotic Bcl-2 was studied to better understand the molecular mechanisms involved. Our data showed that BLM treatment resulted in a progressive upregulation of Caspase-3 protein and downregulation of Bcl-2, but BV showed little expression of Caspase-3 with no significant effect on Bcl-2. There are conflicts in previous studies regarding differences in the gene expression between normal cells and cancerous cells. BV induced apoptosis in a calcium-dependent and caspase-independent pathway in human melanoma A2058 cells but not in normal skin fibroblast Detroit 551 cells [52]. Also, Ip et al. [50] confirmed that BV induced cell cycle arrest and apoptosis in human cervical epidermoid carcinoma Ca Ski cells through both caspase-dependent and caspase-independent pathways. However, Lee et al. [38] noted that the cause of this conflict may be the fact that BV possesses selective cytotoxic properties in both normal and cancerous cells.

Collectively, the results of this study suggested that BV enhanced BLM-killing potential in noncancerous cells, but these cellular events were not accompanied by the activation of the apoptotic machinery or genotoxic alterations. Hence, in view of our results, BV can be used to improve the efficacy of customary chemotherapeutic agents and alleviate the danger of secondary malignancies.
